# Emergence of a virulent porcine reproductive and respiratory syndrome virus (PRRSV) 1 strain in Lower Austria

**DOI:** 10.1186/s40813-016-0044-z

**Published:** 2016-11-01

**Authors:** Leonie J Sinn, Eva Klingler, Benjamin Lamp, Rene Brunthaler, Herbert Weissenböck, Till Rümenapf, Andrea Ladinig

**Affiliations:** 1 Institute of Virology, Department of Pathobiology, University of Veterinary Medicine Vienna, Veterinaerplatz 1, 1210 Vienna, Austria; 2 Vetpraxis Hegerberg, 3072 Kasten 25, Austria; 3 Institute of Pathology and Forensic Veterinary Medicine, Department of Pathobiology, University of Veterinary Medicine Vienna, Veterinaerplatz 1, 1210 Vienna, Austria; 4 Clinic for Swine, Department for Farm Animals and Veterinary Public Health, University of Veterinary Medicine Vienna, Veterinaerplatz 1, 1210 Vienna, Austria

**Keywords:** Porcine reproductive and respiratory syndrome virus (PRRSV), Austria, Field isolate, Acute outbreak

## Abstract

**Background:**

In spring 2015, an outbreak of porcine reproductive and respiratory syndrome (PRRS) struck Lower Austria caused by a PRRS virus (PRRSV) strain spreading rapidly among both previously PRRSV negative and vaccinated pig herds. This case report describes the first well-documented emergence of the PRRSV strain responsible for this outbreak.

**Case presentation:**

A PRRSV seronegative piglet-producing farm in Lower Austria encountered losses in foetuses and suckling piglets of up to 90 %; clinical signs in sows and nursery piglets included fever and reduced feed intake. Additionally, high percentages of repeat breeders and losses of up to 40 % in nursery piglets occurred. An infection with PRRSV was suggested by the detection of antibodies by enzyme linked immunosorbent assay and confirmed by quantitative real time PCR. The underlying PRRSV strain, termed AUT15-33, was isolated by passage on porcine alveolar macrophages, partially sequenced (ORF2-7) and grouped as PRRSV-1, subtype 1. In phylogenetic analysis of the genome region coding for the structural proteins, ORF2-7, AUT15-33 clustered with Belgian strains but identities were as low as 88 %. In contrast, analysis of ORF7 sequences revealed a close relationship to Croatian strains from 2012 with an identity of 94 – 95 %.

**Conclusions:**

In the year following the outbreak, the same PRRSV strain was identified repeatedly in different regions of Austria. It can be speculated that the new strain has novel advantageous properties.

## Background

Porcine reproductive and respiratory syndrome virus (PRRSV) is one of the most economically important viruses affecting the global swine industry. PRRSV is a small, enveloped virus with a single-stranded RNA genome of positive polarity, which is grouped in the family *Arterivirdae* [1–3]. Due to the high degree of genetic diversity, PRRSV was recently divided into two species: PRRSV-1 (formerly European genotype 1) and PRRSV-2 (formerly North American genotype 2) [4, 5]. Extensive genetic differences do exist not only between but also within the two species leading to the sub-classification into at least three European subtypes [6]. Depending on the strain great differences exist, for example in the ability of propagation in different cell lines in vitro [1, 7] or in the pathogenicity in vivo [8–10].

Clinical presentation of PRRS varies greatly between herds and is influenced by genetic and virulence differences of PRRSV isolates, host immune status, host susceptibility, concurrent infections and other management factors [11]. Typical clinical signs of PRRS in nursery and grow/finishing pigs include respiratory signs and reduced growth performance [1, 12]. Of particular importance are secondary and concomitant infections since PRRSV was shown to have an additive or synergistic effect with other bacteria and viruses [13–15]. Reproductive disease associated with PRRSV is characterized by abortions, early farrowings, foetal death and the birth of weak, congenitally infected piglets resulting in elevated pre-weaning mortality [16–18]. Highly pathogenic PRRSV strains have been described for both PRRSV-1 (strain Lena, subtype 3 [19]) and PRRSV-2 (atypical PRRS caused by strains with a characteristic deletion in nonstructural protein 2 [20]). They are characterized by high fever and high mortality rates in pigs of all age groups.

Diagnostic methods include e.g., virus isolation, histologic staining techniques and reverse transcription polymerase chain reaction (RT-PCR), which is most commonly used for routine diagnostics. For virus isolation PRRSV can be grown on primary porcine alveolar macrophages (PAM), which are obtained from lungs of PRRSV-free pigs. The only PRRSV-permissive cell lines, MA-104 or its clone MARC-145, rarely support the spontaneous growth of PRRSV-1 strains. The detection of PRRSV-specific antibodies is most commonly performed by enzyme-linked immunosorbent assay (ELISA). Control measures for PRRSV include the prevention of virus introduction into herds by applying strict biosecurity standards and the regular use of modified live vaccines (MLV).

Austria is a small country in Central Europe that directly neighbours eight nations. Due to the geopolitical situation, Austria is bridging the trade of western and eastern, northern and southern European countries. Import or transit of animals or animal products implies the risk of acquiring virus diseases of livestock and hence Austria might act as a sentinel. PRRSV is considered endemic in Austria (as in most other countries) although detailed epidemiological data is limited [21, 22]. Available sequence information on Austrian PRRSV strains include several ORF5 and ORF7 sequences and two full-length genomes of PRRSV isolates [23–26], all belonging to PRRSV-1 subtype 1.

Here we describe the first well-documented case of a cluster of acute outbreaks of PRRS in Lower Austria in spring 2015. Losses on the presented piglet-producing farm in Lower Austria went up to 90 % in one farrowing batch. The underlying virus strain, named AUT15-33, showed a high similarity with Croatian strains in ORF7 and proved its epidemic potential in the year following the outbreak by spreading to other regions in Austria.

## Case presentation

### Anamnesis and physical findings

The case herd was kept on a family owned, piglet producing farm located in Lower Austria, which was known to be free of PRRSV since ten years based on routine serological testing of sows and nursery piglets performed twice per year. The farm was producing piglets with 80 sows in a 3-week batch-farrowing interval; suckling period was about 28 days. At the end of the nursery period, 30 kg piglets were sold to one finishing farm. Gilts were bought from one multiplier herd in Lower Austria.

**Fig. 1:**
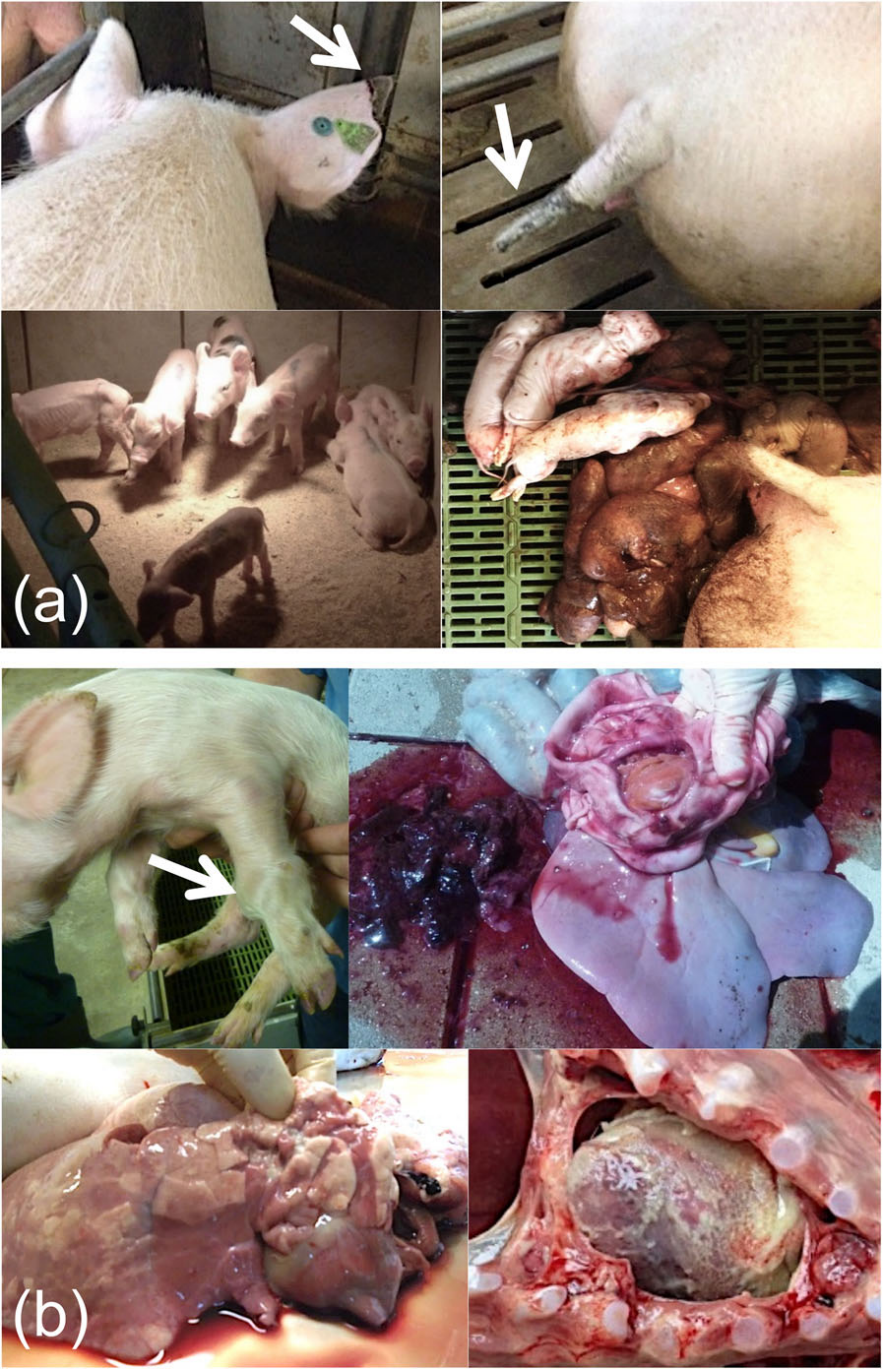
(**a** + **b**) Clinical signs in sows (**a**) and nursery piglets (**b**). **a** Affected sows showed cyanosis on ears and tail (green arrows) and gave birth to dead or weak piglets. **b** Piglets in nursery suffered from various clinical signs including swollen joints, stomach ulcers/gastritis, pericarditis and pneumonia

Clinical problems in the herd started in the beginning of April 2015; sows had reduced to completely absent feed intake and high fever (>41 °C). Additionally, cyanosis on ears and tail was visible in individual sows, which developed into ear and tail necrosis later in the course of disease (Fig. 1a). The herd veterinarian decided to treat the sows with acetylsalicylic acid due to the suspected diagnosis of an influenza virus infection. However, clinical signs did not improve and reproductive disorders started to occur when the next batch of sows farrowed mid-April. In the particular farrowing batch, around 50 % of piglets were born dead and another 40 % of piglets died within the first days of live. Only eleven piglets were weaned from nine sows and all showed reduced growth performance (Fig. 1a). Two sows farrowed ten days delayed and were less severely affected; a total of 20 piglets could be weaned from those two sows, with five piglets clearly retarded in growth. A similar clinical picture was observed in the next farrowing batch; 38 piglets from ten litters survived the first two weeks of life but were clearly reduced in their growth performance. Interestingly, all sows farrowed at their due date with the exception of one sow which farrowed two days early and delivered dead piglets only.

Reproductive problems also occurred in early gestation leading to a return to oestrus rate of 60 % in sows bred beginning of April and 40 % in sows bred end of April.

Clinical problems in the nursery started end of April in piglets about five weeks of age (one week after weaning). Piglets showed fever, swollen joints and lameness, severely reduced feed intake and runting (Fig. 1b). About 40 % of piglets from this age group died or had to be euthanized. Antibiotic treatment with various antimicrobials (including amoxicillin, colistin sulphate, ceftiofur, etc.) did not improve the situation. Clinical signs spread slowly throughout the nursery. Next, the oldest group of piglets close to delivery to the finishing farm was affected. Piglets showed signs of diarrhoea, reduced growth performance, anaemia and about 10 % of piglets died in this age group. The least affected group of piglets was the age group in mid nursery; here piglets showed mild respiratory signs like coughing and dyspnoea, but no losses occurred.

**Fig. 2:**
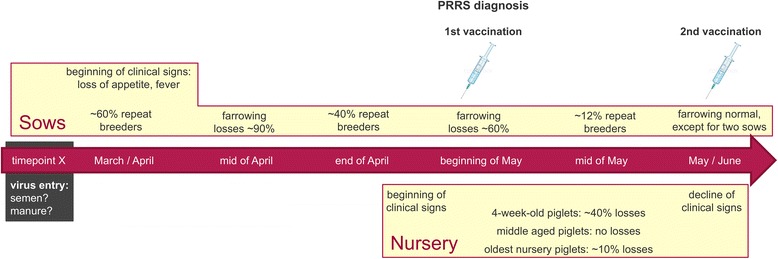
Time course of infection. Observed clinical problems are summarized in a timely manner

A summary of clinical observations including a time course of events is illustrated in Fig. 2.

### Diagnostic methods and laboratory findings

**Fig. 3:**
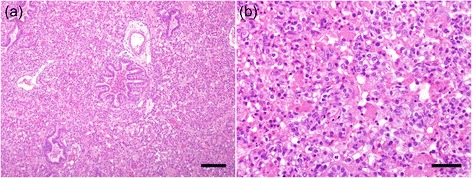
(**a** + **b**) Histological lung lesions. **a** Interstitial pneumonia and catarrhalic to purulent bronchopneumonia with severe atelectasis (bar length 150 μm). **b** Intralobular interstitial pneumonia including hyperplasia of type II pneumocytes and necrotic cells in the alveolar lumen (bar length 60 μm)

First samples for diagnostics were taken end of April from sows, which started to show clinical signs at least two weeks prior to sampling. Blood samples were taken from nine sows for serological investigation. Antibodies against PRRSV were detected by ELISA in sera of all sows. To investigate concomitant infections in nursery piglets, two piglets from the most severely affected age group (around seven weeks old at the time of submission), showing poor body condition, enlarged inguinal lymph nodes and respiratory signs like coughing were selected by the herd veterinarian and submitted for necropsy and further diagnostics to the University of Veterinary Medicine Vienna. Post mortem investigations found poor retraction of the lung and consolidation of the cranio-ventral areas. A catarrhal enterocolitis was diagnosed in one piglet. Histologically, atelectasis with intralobular, interstitial pneumonia including hyperplasia of type II pneumocytes, as well as a catarrhalic to purulent bronchopneumonia were seen in the lungs of both pigs (Fig. 3). Bacterial isolation was only performed on organs showing pathological alterations (intestines and lung). While in intestinal samples no pathogenic bacteria could be detected, *Staphylococcus hyicus* could be isolated from the lung of both pigs by conventional bacteriological culture. To exclude an involvement of PCV2 an in situ hybridization (ISH) on inguinal lymph nodes was performed. No histologic lesions were found in the lymph nodes and no PCV2 was detected by ISH.

Due to the wet summer of 2014 elevated mycotoxin levels were expected for corn fed in 2015. Mycotoxin analyses revealed increased levels of deoxynivalenol (DON, 5040 μg/kg) and zearalenone (ZEA, 851 μg/kg). The proportion of corn in feed was about 16.5 % for sows and 30 % for piglets.

**Fig. 4:**
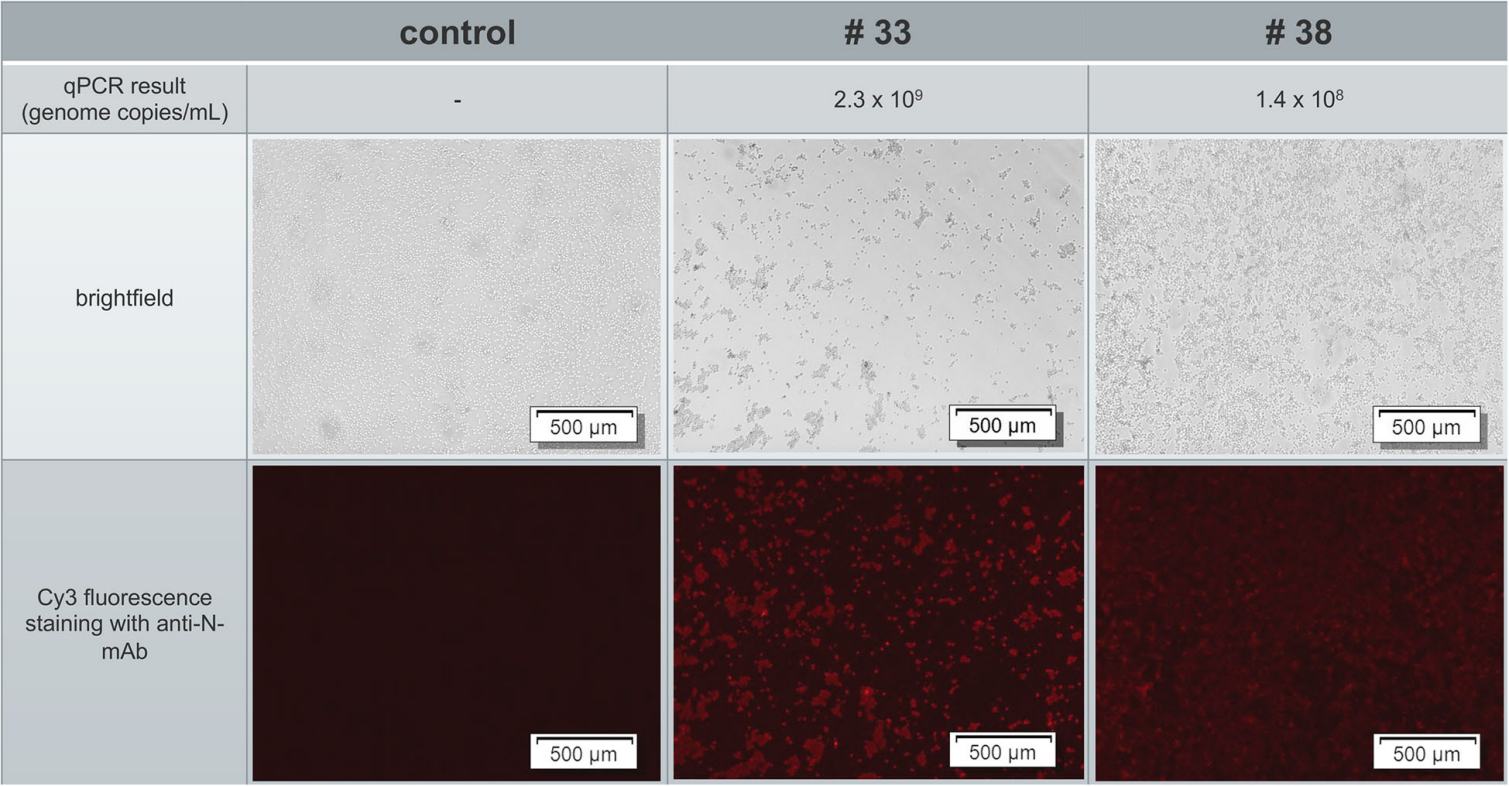
Virus isolation of field strain AUT15-33. Serum of nine acutely affected nursery piglets was inoculated on porcine alveolar macrophages (PAM). After two days cells were fixed with methanol-acetone and immunofluorescence stained with an anti-PRRSV-N-protein monoclonal antibody (kindly provided by A. Saalmüller, Vienna) [39] and goat-anti-mouse conjugated with Cy3 as a secondary antibody. Varying cytopathic effects were seen and a PRRSV-specific immunofluorescence staining was detected (serum samples #33 and 38 are shown exemplarily)

Since the clinical course of infection was unusually severe for Austrian conditions and reports about PRRS outbreaks in the same region accumulated, the PRRSV strain was further characterized. Blood samples were collected from nine nursery piglets showing acute clinical signs like fever and reduced alertness in order to isolate the virus and perform RT-PCR and sequencing. To this end naïve primary cells (PAM) and the cell line MARC-145 were inoculated with serum from each piglet. A PRRSV-specific immunofluorescence staining of PAM (Fig. 4) but not of MARC-145 cells could be detected after two days. This finding was confirmed after passaging the supernatant on new PAM and MARC-145 cells (data not shown). In accordance, all nine serum samples were tested highly positive when using a commercial PRRSV quantitative RT-PCR (qRT-PCR) Kit with genome copy numbers between 1.4 x 10^8^ and 4.3 x 10^9^ (Table 1). In conclusion, a new PRRSV strain, named AUT15-33, could be isolated from serum of piglets showing acute illness on primary PAM.

**Table 1:** Viral load in serum of acutely affected nursery piglets quantified by a commercial PRRSV qRT-PCR Kit (TaqMan®NA and EU PRRSV Reagents, Ambion, Carlsbad, USA)

Sample ID	CT-value	PRRSV RNA copies/mL serum
32	16.02	2.2 x 10^9^
33	15.99	2.3 x 10^9^
34	18.41	4.8 x 10^8^
35	16.02	2.2 x 10^9^
36	17.26	1.1 x 10^9^
37	14.99	4.3 x 10^9^
38	20.39	1.4 x 10^8^
39	18.98	3.3 x 10^8^
40	17.51	8.6 x 10^8^

Total RNA was extracted from PAM five days after inoculation and used for RT-PCR. Two primer pairs (PRRSV-I-F 5’-GACCATATCTGCAACCTGAGAC-3’/PRRSV-I-R 5’- CAATTTGTGAGAACATCTCATATC-3’ and PRRSV-II-F 5’- CTTTTCTACGCCTCAGAAATGAG-3’/PRRSV-II-R 5’- TTTGGATCCAACGTTTTTTTTTTTTTTTTTTTTTT-3’) were applied to generate overlapping PCR amplicons, which covered the whole genome region encoding the structural proteins (ORF2-7). The DNA fragments were subjected to gel electrophoresis, purified and sequenced by a commercial laboratory (Eurofins Genomics, Ebersberg, Germany). The determined ORF2-7 genome sequence of AUT15-33 was submitted to GenBank (KU494019).

**Fig. 5:**
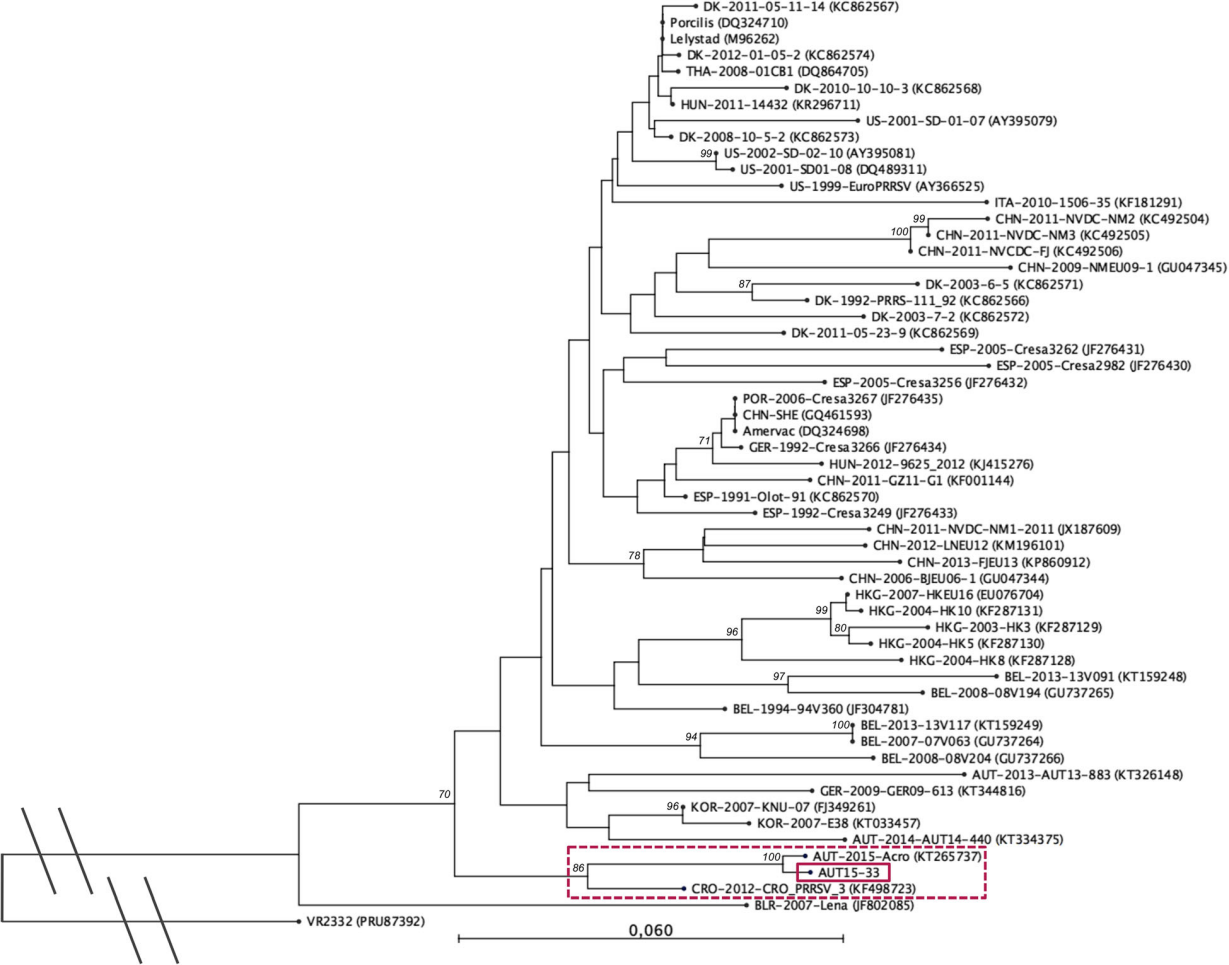
Phylogenetic analysis based on ORF7 nucleotide sequences of 54 PRRSV-1 strains and PRRSV-2 prototype VR2332 as an out-group. The PRRSV strain presented in this study is marked with a solid box and the associated sub-tree is highlighted with a dotted box. The tree was constructed with the software CLC Sequence Viewer 7.6 (CLCBIO, Aarhus, Denmark) using the neighbour joining method with the numbers at the nodes representing bootstrap values in % of 1000 replicates. Scale bar: number of substitutions per site

**Fig. 6:**
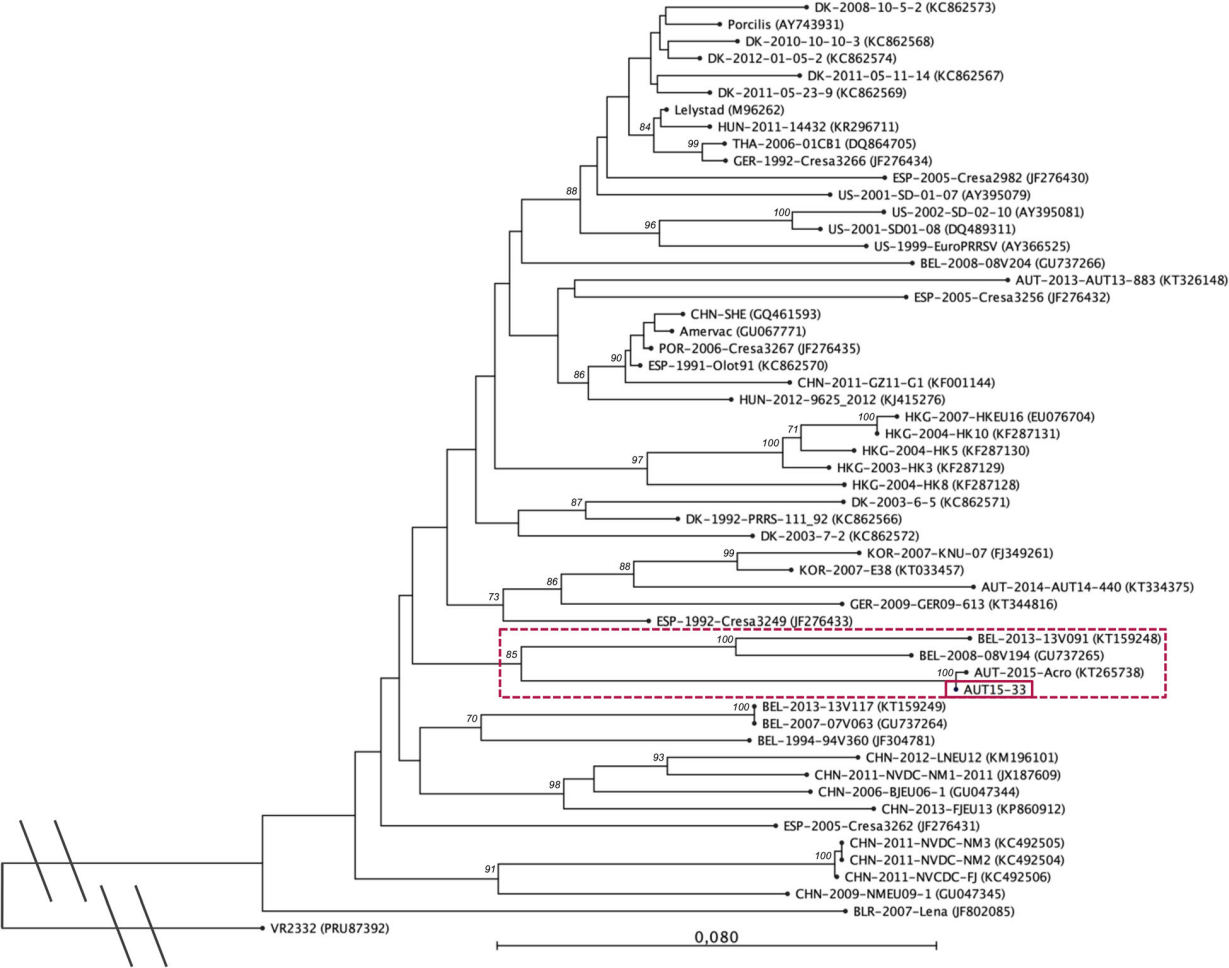
Phylogenetic analysis based on ORF5 nucleotide sequences of 53 PRRSV-1 strains and PRRSV-2 prototype VR2332 as an out-group. The PRRSV strain presented in this study is marked with a solid box and the associated sub-tree is highlighted with a dotted box. The tree was constructed with the software CLC Sequence Viewer 7.6 (CLCBIO, Aarhus, Denmark) using the neighbour joining method with the numbers at the nodes representing bootstrap values in % of 1000 replicates. Scale bar: number of substitutions per site

**Fig. 7:**
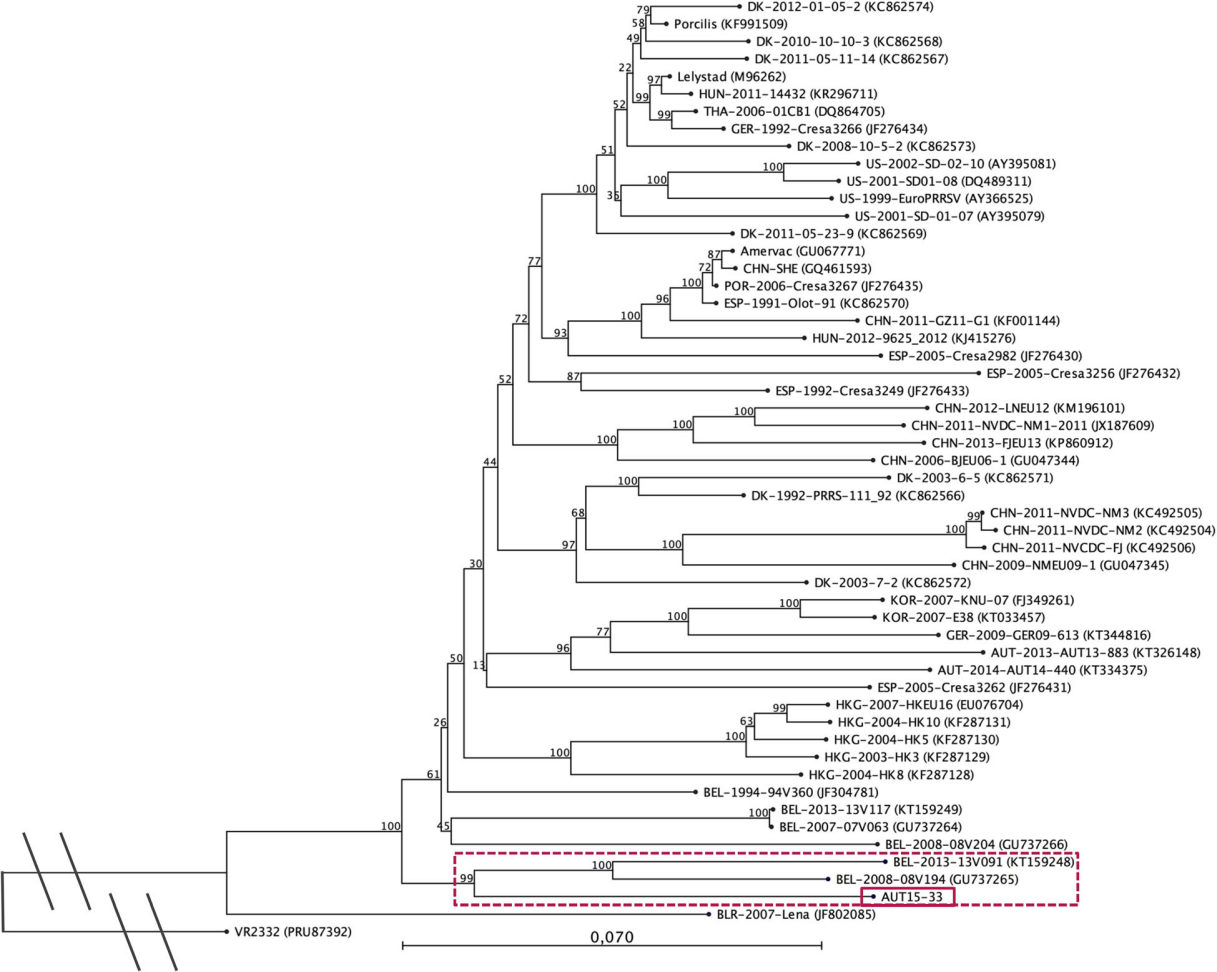
Phylogenetic analysis based on ORF2-7 nucleotide sequences of 52 PRRSV-1 strains and PRRSV-2 prototype VR2332 as an out-group. The PRRSV strain presented in this study is marked with a solid box and the associated sub-tree is highlighted with a dotted box. The tree was constructed with the software CLC Sequence Viewer 7.6 (CLCBIO, Aarhus, Denmark) using the neighbour joining method with the numbers at the nodes representing bootstrap values in % of 1000 replicates. The ORF2-7 sequence of AUT15-33 was submitted to GenBank with provisional entry KU494019. Scale bar: number of substitutions per site

Initial phylogenetic analysis and identity calculations were carried out with NCBI’s Basic Local Alignment Search Tool for nucleotides (BLASTn). All PRRSV-1 strains with full ORF2-7 sequences deposited in GenBank as well as the two closest neighbours - as determined by NCBI BLASTn - were used to construct the phylogenetic trees. For ORF5 and ORF7, a nucleotide identity >99 % was found with the Austrian PRRSV strain ‘Acro’ (KT265737), which has been submitted to GenBank in July 2015 and very likely originates from the same PRRS outbreak. In the phylogenetic tree based on ORF7 both AUT15-33 and Acro cluster with several Croatian strains from 2012 [27], one of them (CRO_PRRSV_3, KF498723) is shown as a representative, but not with other current Austrian PRRSV sequences from 2013 and 2014 [26] (Fig. 5). The identity with the Croatian strains ranges from 94 to 95 %. Due to missing sequence data for the Croatian strains, the relatedness to them could not be investigated further. The phylogenetic tree built with ORF5 sequences shows the new Austrian isolate clustering with the Belgian strains 13 V091 and 08 V194 [28, 29] (Fig. 6). However, the nucleotide identity between these and AUT15-33 is only 86 %, the same as with the PRRSV-1 prototype strain Lelystad virus (LV). A similar pattern is seen in a phylogenetic tree based on a larger sequence part (3.2 kb) including the genome region coding for the structural proteins (ORF2-7) (Fig. 7). Here the identity of AUT15-33 to both Belgian strains mentioned above and to LV is 88 %.

### Further steps and outcome of case

When laboratory tests confirmed an infection with PRRSV, the herd veterinarian decided to perform mass vaccination of all pigs on site using a PRRSV-1 MLV (Porcilis® PRRS, MSD Animal Health, Boxmeer, Netherlands). Pigs were vaccinated twice: the first vaccination was performed in the first week of May, the second vaccination four weeks later. Additionally, the mycotoxin content in feed was reduced by lowering the percentage of corn within the diet.

The clinical situation in sows improved during the course of May; about 88 % of sows inseminated mid-May became pregnant. With the exception of two sows, which delivered dead piglets only, no further reproductive problems occurred in the group of sows farrowing end of May. In nursery piglets an improvement of clinical signs was not seen until end of May in the most severely affected group of piglets. The age group at mid-nursery never experienced severe clinical signs and no losses occurred in this group.

The route of PRRSV introduction into the case herd could not be identified. Gilts were unlikely the source of PRRSV introduction because a detailed screening for PRRSV antibodies in the multiplier herd was negative and no other clients of that herd experienced problems with PRRSV. Since the case herd shared boars with a commercial piglet producing farm, which experienced an outbreak with PRRSV, this was the most likely route of PRRSV introduction into the case herd. Also, manure-pumping equipment was shared between the case herd and other farms. Manure was pumped from the case herd about two weeks prior to the outbreak and the equipment could have been a potential route of PRRSV introduction. Because several farms in the surrounding area experienced PRRS outbreaks at the same time, other routes of infection like aerosol transmission, people or vehicles acting as vectors for the virus cannot be excluded.

Ever since the first outbreaks with PRRSV AUT15-33 have been identified, the particular virus strain has been found in various regions of Austria. The intramural diagnostic laboratory alone could confirm the same strain on 16 additional farms. More recently, similar PRRSV isolates were found in pig farms in Germany (AL, personal communication).

## Conclusions

The acute outbreak of PRRS in the case herd was characterized by massive losses in both suckling and nursery piglets, indicating a PRRS outbreak rather unusual for Austria with high economic losses. This is of particular importance since it is the first well-documented case of a cluster of outbreaks in Lower Austria in 2015 most likely caused by the same virus strain.

Before the PRRS outbreak, despite the high levels of mycotoxins in the feed, performance of sows and piglets in the case herd was good with losses below 2 % and minimal antimicrobial treatment in nursery piglets. The severe clinical signs and high losses in nursery pigs suggest that PRRSV is important as a primary pathogen as well as an immunomodulating factor causing secondary infections in pigs (e.g., *Staphylococcus hyicus* in this case).

As an immediate intervention strategy, the herd veterinarian decided to perform mass vaccination with MLV. On the one hand, it has to be considered that an additional virus strain was introduced into the herd by the use of MLV. Modified vaccine virus is known to behave similar to field virus in regards to transmission, persistence, transplacental infection, shedding and time required to induce immunity. Also, field reports raised the concern of reversion to virulence of attenuated vaccine virus [30]. On the other hand, PRRSV MLVs were proven to be able to reduce clinical signs and lesions after PRRSV infection [31–35]. Additionally, MLV reduced wild-type virus shedding from infected populations to the environment [36]. Also, MLV could be effectively used to eliminate PRRSV from herds by homogenizing the immune status of the animals since it was known that PRRSV does not persist in immune populations [37, 38]. Considering the slow spread of the virus within the case herd, the use of MLV might have been beneficial for pigs that have not yet been naturally infected.

The isolation of PRRSV field strains in cell culture is an important tool to further characterize virus strains because virus growth in cell culture allows sequencing of larger genomic regions. For phylogenetic analysis and recombination studies, both crucial to monitor PRRSV epidemiology and to evaluate the effectiveness of current vaccine strategies, it is essential to sequence parts of the genome larger than ORF5 or ORF7. These regions have been widely used in routine diagnostics but account for only 4 and 2.5 % of the whole genome, which limits their significance for phylogenetic studies, especially, if only one of them is determined. ORF7 of AUT15-33 shows a high degree of similarity to Croatian strains from 2012, a finding that cannot be confirmed in analysis of larger genomic regions because for these strains only ORF7 sequences exist (J. Prpić, personal communication) [36]. When analysing ORF2-7 or ORF5, AUT15-33 clustered with strains from Belgium but identities were not higher than 88 %, excluding a close relationship. The same identity occurs to the prototype strain LV, which clusters totally different in a phylogenetic tree. The reason is that phylogenetic analysis is not based on identity calculations but on algorithms calculating evolutionary development. Sequences that cluster in the same lineage and show a high sequence identity to AUT15-33 could fill the evolutionary gap between the Belgian strains and AUT15-33 but are missing. Surprisingly there is no close relationship to Austrian strains previously detected (e.g., to strains from 2013 to 2014 [26] or earlier [24, 25]). It is questionable whether the lack of close relatives is due to the few sequences available or to a novel introduction to Austria. We favour the idea that the strain was newly introduced to Austria because the outbreak of AUT15-33 occurred simultaneously on many farms and caused high losses, indicating naïve herds. With regard to the high similarity to the Croatian strains an origin in south-eastern Europe seems possible but as long as there is limited data available on PRRSV strains in Austria and neighbouring countries, it can only be speculated about potential introduction routes of AUT15-33.

Availability of sequence information from strains currently circulating in the field is not only important for epidemiology but also for evaluation of diagnostic methods. Since PRRSV is a RNA virus and therefore has a high mutation frequency, sequencing of primer binding regions is key to ensure that current field strains are still detected by standard PRRSV diagnostics. Therefore, extensive sequence information given in this case report about the Austrian field isolate AUT15-33 is highly valuable.

An advantage of virus isolation is the availability of these isolates for further research, for molecular studies as well as for animal trials to evaluate vaccine efficacy. This is especially important since AUT15-33 has spread widely throughout Austria in the year following the outbreak described here, affecting not only PRRSV naïve but also vaccinated herds. The rapid spread of this strain in the field indicates advantages compared to other PRRSV-1 strains. Whether underlying mechanisms include e.g., a faster replication, an easier entry into target cells, a more effective down regulation of innate immunity or stronger immune evasion properties remains to be elucidated in future experiments.

In this case report, we describe a PRRSV-1 outbreak in a piglet-producing farm that for Austrian standards was characterized by unusually severe reproductive losses and losses in nursery piglets. We link this information to the genetic and epidemiologic background of the virus strain and the in vitro characteristics of the virus isolate. In our hands this combination of clinical information with molecular biological analysis is key to modern PRRSV management.

## Abbreviations

DON: Deoxynivalenol
ELISA: Enzyme-linked immunosorbent assay
ISH: In situ hybridization
LV: Lelystad virus
MLV: Modified live vaccines
ORF: Open reading frame
PAM: Porcine alveolar macrophages
PCV2: Porcine Circovirus type 2
PRRS: Porcine reproductive and respiratory syndrome
PRRSV: Porcine reproductive and respiratory virus
qRT-PCR: quantitative reverse transcription polymerase chain reaction
RT-PCR: Reverse transcription polymerase chain reaction
SIV: Swine influenza virus
ZEA: Zearalenone
